# A rhein-integrated hydrogel dressing with antibacterial, antioxidant and pH-responsive colorimetric properties

**DOI:** 10.1039/d6ra03047e

**Published:** 2026-05-22

**Authors:** Miaoshen Kou, Deng Wang, Jiawei Zhang, Fangzheng Yu, Yang Yuan, Chen Wang, Jiale He, Zheng Zhao, Yichao Liu

**Affiliations:** a State Key Laboratory of Advanced Technology for Materials Synthesis and Processing, Wuhan University of Technology Wuhan 430070 China zhengzhao@whut.edu.cn; b Respiratory and Critical Care Medicine Department of Ezhou Central Hospital Ezhou City 436000 China; c Sanya Science and Education Innovation Park, Wuhan University of Technology Sanya 572000 China; d Department of Dermatology, Zhongnan Hospital of Wuhan University, Wuhan University Wuhan 430071 China yichaoliu619@whu.edu.cn

## Abstract

Integrating bioactive function with wound-status indication remains a major challenge in the design of hydrogel dressings for complex wound management. Most pH-responsive hydrogels still rely on separate bioactive and sensing components, leading to increased compositional and fabrication complexity and highlighting the need for unified platforms based on a single bioactive molecule with intrinsic pH responsiveness. In this work, a multifunctional SF/HA/Rhe hydrogel dressing was developed by incorporating rhein (Rhe), a plant-derived small molecule, into a silk fibroin/hyaluronic acid (SF/HA) matrix. Rhe served as a single functional component that simultaneously endowed the hydrogel with antibacterial, antioxidant and pH-responsive colorimetric properties. The optimized SF/HA/Rhe hydrogel achieved antibacterial rates above 94% against *Staphylococcus aureus*, *Escherichia coli* and methicillin-resistant *Staphylococcus aureus* (MRSA). It also exhibited strong antioxidant activity, with an ABTS radical scavenging rate of 87.22% and effective reduction of intracellular ROS accumulation. In addition, the hydrogel showed a distinct pH-dependent colorimetric response over the wound-relevant pH range of 5.5–7.5, allowing visual indication of local pH variations. The SF/HA/Rhe hydrogel further displayed favorable mechanical performance and biocompatibility, with cell viability above 90% and hemolysis below 5%. Overall, these findings provide a feasible strategy for multifunctional hydrogel dressings with integrated antibacterial, antioxidant and visual pH monitoring.

## Abstract 2

The novel SF/HA/Rhe hydrogel exhibits antibacterial, antioxidant and pH-responsive colorimetric functions and has potential application as multifunctional wound dressing for visual monitoring.

## Graphical abstract

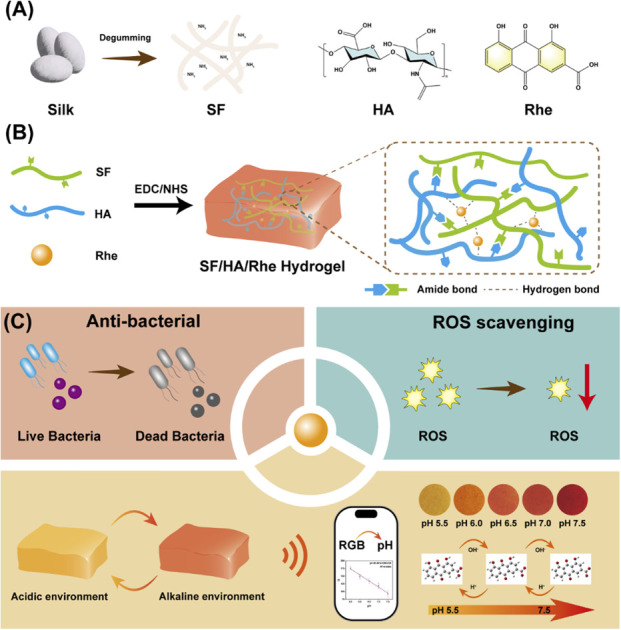

## Introduction

In recent years, natural polymer-based hydrogels have attracted considerable attention in wound repair owing to their high water-retention capacity, good biocompatibility and biodegradability.^1–3^ Their hydrated three-dimensional networks can absorb excess wound exudate and maintain a moist local microenvironment, which is beneficial for wound repair.^4–6^ In native tissues, the extracellular matrix (ECM) provides structural support and a suitable biological microenvironment for cell adhesion, migration, proliferation and other physiological processes. Fibrous proteins and polysaccharides are major constituents of the ECM.^7,8^ Therefore, hydrogels constructed from these biomacromolecules can mimic key compositional and microenvironmental features of the native ECM, making them promising platforms for wound repair and regeneration.

Hyaluronic acid (HA), a non-immunogenic glycosaminoglycan abundant in the ECM, is widely used in biomaterials because of its excellent hydrophilicity, biocompatibility and ability to maintain a hydrated microenvironment.^9,10^ It can also interact with multiple cellular receptors and thereby contribute to cell migration, proliferation and tissue repair. Nevertheless, HA-based hydrogels often suffer from insufficient mechanical strength and rapid degradation, while their highly hydrophilic and polyanionic characteristics may be unfavorable for cell adhesion.^11^ Silk fibroin (SF) is a natural fibrous protein derived from silkworm silk. Its good biocompatibility, excellent mechanical properties and favorable cell adhesion make it an attractive material for tissue engineering applications.^12,13^ In addition, the mechanical properties and degradation behavior of SF hydrogels can be readily tuned.^14^ Therefore, combining SF with HA provides a rational strategy for constructing composite hydrogels that better reflect the composition and functions of the ECM. Such hydrogels can offer improved mechanical support and structural stability while maintaining favorable biocompatibility for tissue repair applications.

An ideal hydrogel dressing should also have antibacterial and antioxidant properties to suppress infection and alleviate oxidative stress.^15,16^ However, SF/HA composite hydrogels may still be insufficient to address bacterial infection and oxidative stress. In recent years, plant-derived small molecules, such as curcumin,^17^ quercetin^18^ and berberine,^19^ have attracted considerable attention in the development of antibacterial hydrogels. Compared with conventional antibiotics, these phytochemicals often exert antibacterial effects through distinct mechanisms. Therefore, they may help reduce reliance on traditional antibiotics and potentially delay the emergence of antibiotic-resistant bacteria. In addition, some of these plant-derived molecules also exhibit antioxidant and anti-inflammatory activities, which further support their potential in infection treatment. Among them, rhein (Rhe), an anthraquinone derivative isolated from Chinese herbal medicine, is particularly attractive for its antibacterial and antioxidant activities.^20,21^ More importantly, the unique molecular structure of Rhe endows it with intrinsic pH-responsive colorimetric behavior, making it particularly attractive for wound-status indication. Wound pH is an informative microenvironmental indicator because it changes dynamically with infection status and healing progression.^22^ In general, infected wounds tend to exhibit a more neutral-to-alkaline microenvironment, whereas a shift toward a more acidic environment is often associated with healing.^23^ Therefore, pH-responsive dressings may serve as useful platforms for visual wound-status indication. Diverse strategies, including pH-indicator dyes,^22^ carbon quantum dots^24^ and pH-sensitive optical fibers,^25^ have been explored for wound pH monitoring. However, many multifunctional hydrogels still rely on these sensing elements as additional modules together with separate antibacterial and antioxidant components, resulting in increased compositional complexity and cumbersome fabrication. Therefore, using SF/HA hydrogels as a carrier platform for Rhe not only helps preserve its bioactivity and pH responsiveness, but also simplifies material design by avoiding the complexity associated with conventional multi-component systems. To the best of our knowledge, no integrated hydrogel has yet been reported in which Rhe serves as a single functional molecule to simultaneously achieve antibacterial, antioxidant and pH-responsive colorimetric functions.

**Fig. 1:**
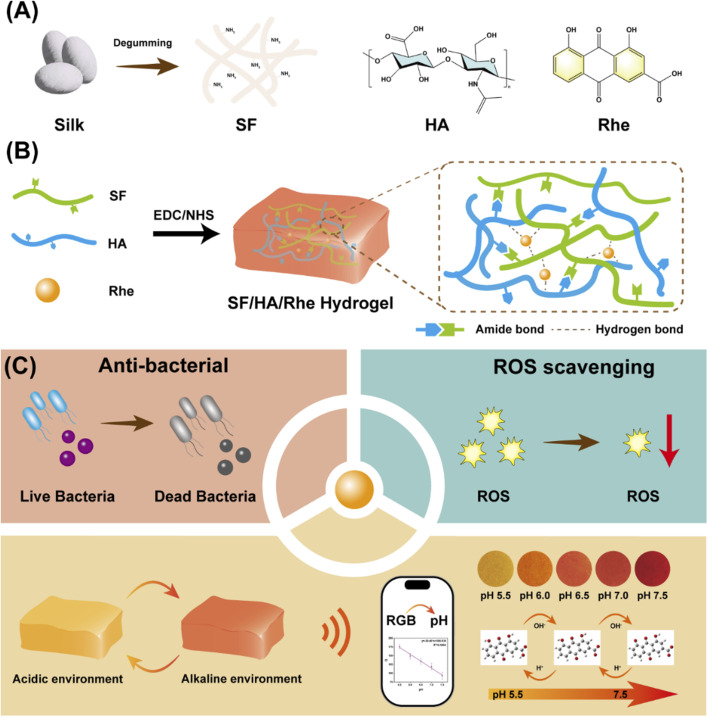
Illustration of the preparation process and multifunctional performance of the SF/HA/Rhe hydrogel: (A) schematic of SF/HA/Rhe hydrogel preparation; (B) schematic of hydrogel formation and network construction; (C) schematic of the antibacterial, antioxidant and pH-responsive colorimetric functions of the hydrogel.

To address the compositional complexity and cumbersome fabrication processes of many existing multifunctional hydrogels, Rhe was introduced as a single plant-derived functional component into an SF/HA hydrogel (Fig. 1). Using a facile cross-linking strategy, a multifunctional hydrogel with antibacterial, antioxidant and pH-responsive colorimetric properties was constructed. The resulting hydrogel was systematically characterized for its physicochemical structure, mechanical performance, swelling and release behavior. Its antibacterial activity, antioxidant capacity, biocompatibility, intracellular ROS-scavenging performance under oxidative stress and pH-responsive colorimetric behavior were further evaluated. This study provides a feasible strategy for constructing multifunctional hydrogels with integrated antibacterial, antioxidant and pH-responsive colorimetric functions.

## Materials and methods

### Materials

Raw silk fibers were purchased from the Zhejiang Silk Market (Huzhou, China). Rhein (Rhe), hyaluronic acid (HA, molecular weight 40–80 kDa), 1-(3-dimethylaminopropyl)-3-ethylcarbodiimide (EDC, ≥98.5% purity), and *N*-hydroxysuccinimide (NHS, ≥98.5% purity) were obtained from MacLean Biochemical Technology Co., Ltd (Shanghai, China). Phosphate-buffered saline (PBS) and DMEM/F-12 medium were purchased from Thermo Fisher Scientific (Shanghai, China). Calcein-AM/propidium iodide (PI), trypsin, and DCFH-DA were obtained from Beyotime Biotechnology (Shanghai, China).

### Preparation of SF

Raw silk was processed according to previously reported methods.^26^ Briefly, 10 g of silk was boiled in a sodium carbonate solution for degumming, thoroughly washed with deionized water and subsequently dried in a laboratory oven set at 60 °C. The dried SF samples were dissolved in a 9.3 M lithium bromide solution, followed by heating for 2 h to yield a SF solution. Subsequent to this step, the resulting solution was transferred into a dialysis bag with a molecular weight cutoff of 13 kDa and dialyzed against deionized water for a total period of 72 h, with frequent water changes. Finally, the dialyzed solution was frozen and lyophilized to yield silk fibroin powder.

### Preparation of SF/HA hydrogels

SF and HA powders were solubilized in deionized water at a mass ratio of 1 : 1, yielding an SF/HA mixed solution with an overall concentration of 3 wt%. 0.6 wt% NHS was added to the mixture, prior to which 1.5 wt% EDC had been introduced for the activation of carboxyl moieties in HA. The mixture was thoroughly stirred at 25 °C until gelation occurred, yielding the SF/HA hydrogel.

### Preparation of the SF/HA/Rhe hydrogels

Rhe powders were first solubilized in deionized water, and the pH of the resultant solution was adjusted to prepare Rhe solutions with concentrations of 5, 10, and 15 mg mL^−1^, respectively. Meanwhile, 10 mL of SF/HA solution was prepared as described above. Subsequently, 1.5 wt% EDC, 1 mL of Rhe solution at different concentrations, and 0.6 wt% NHS were sequentially added to the SF/HA solution. The mixture was stirred thoroughly at 25 °C to obtain SF/HA/Rhe hydrogels, which were designated as SF/HA/Rhe_5_, SF/HA/Rhe_10_, and SF/HA/Rhe_15_, respectively.

### Characterization

Fourier transform infrared (FT-IR) spectroscopy was employed to characterize the chemical constituents of Rhe, SF, HA, and hydrogel specimens. Each sample was ground with KBr and pressed into pellets, which were subjected to FT-IR spectroscopy scanning over the wavenumber range of 400–4000 cm^−1^. The UV-vis absorption spectra of Rhe solutions were measured over the wavelength range of 300–800 nm with a UV-vis spectrophotometer (UV-2550, Shimadzu, Japan).

### Water content, porosity and swelling performance

Hydrogel samples with a wet weight (*W*_w_) were dried in a vacuum freeze-dryer for 48 h to obtain the dry weight (*W*_d_). The water content was calculated as:1
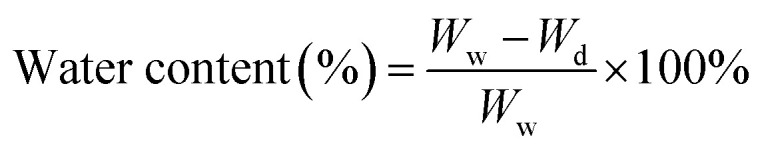


For the purpose of evaluating swelling behavior, pre-dried hydrogel samples (*W*_0_) were immersed in PBS under room temperature conditions. At predetermined time intervals, the swollen hydrogels were retrieved, blotted gently to remove surface moisture, and weighed to record their mass (*W*_*t*_). The swelling rate was computed as follows:2
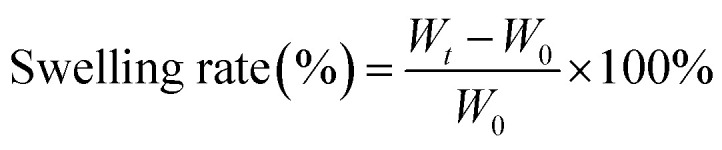


The porosity of the hydrogels was determined by the solvent displacement method described previously.^27^ Freeze-dried hydrogel samples with a cylindrical shape were weighed and the initial mass was recorded as *W*_0_. The diameter and height of each sample were measured using a vernier caliper, and the volume (V) was calculated accordingly. The samples were then immersed in anhydrous ethanol. After ethanol had completely penetrated the pores, the samples were removed, gently blotted with filter paper to remove residual ethanol from the surface, and weighed again to obtain *W*_1_. The porosity (P) of the samples was calculated according to the following equation:3
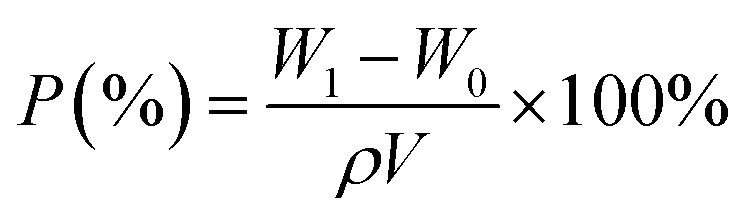
where *P* is the density of anhydrous ethanol.

### Mechanical and rheological properties

#### Compression test

The compressive properties of SF/HA and SF/HA/Rhe hydrogels were evaluated using a universal testing machine. Cylindrical samples (20 mm diameter × 15 mm height) were subjected to uniaxial compression under a constant loading rate of 10 mm min^−1^. The compressive strength was defined as the stress at 60% strain.

#### Rheological test

Rheological characterization was conducted with a rotational rheometer (HAAKE MARS 40, Germany) at 25 °C. Strain amplitude sweep tests were executed at a constant frequency of 1 Hz across a strain interval of 1–1000% to quantify the storage modulus (*G*′) and loss modulus (*G*″) of the hydrogels.

### DFT calculations

Geometry optimization and vibrational frequency analyses of Rhe were performed using Gaussian software. The calculations were performed with the CAM-B3LYP functional.

### *In vitro* antibacterial assay

The antibacterial activity of SF/HA/Rhe hydrogels was evaluated using an agar plate assay.^28,29^ Briefly, 1 g of sterilized hydrogel specimens were individually immersed in bacterial suspensions of *Staphylococcus aureus* (*S. aureus*), *Escherichia coli* (*E. coli*), or methicillin-resistant *Staphylococcus aureus* (MRSA). The resultant mixtures were then incubated in a shaking incubator at 37 °C with an agitation speed of 120 rpm for 24 h. Upon completion of incubation, the bacterial suspensions were subjected to serial dilution with PBS under a laminar flow hood. Subsequently, 100 µL aliquots of the diluted bacterial suspensions were uniformly spread across the entire surface of sterile agar plates using a sterile spreader. The agar plates were inverted and incubated at 37 °C for 12 h, after which colony images were captured to assess bacterial growth status and quantify the bacterial survival rate.

### Hemolysis assay

The *in vitro* hemolytic activity of the SF/HA/Rhe hydrogels was evaluated using red blood cells (RBCs). Briefly, 2.0 mL of diluted RBC suspension was added to each centrifuge tube, followed by the addition of hydrogel samples (100 mg). Deionized water and PBS were used as the positive and negative controls, respectively. The tubes were incubated at 37 °C for 3 h and then centrifuged for 10 min to collect the supernatants. The absorbance of the supernatants was measured at 545 nm using a microplate reader. The hemolysis rate was calculated as follows:4
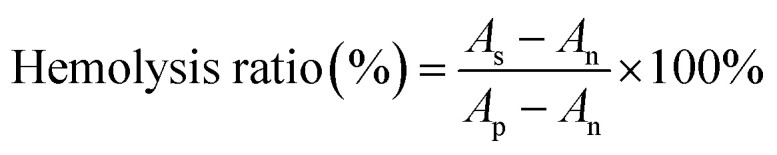
where *A*_s_, *A*_n_, and *A*_p_ represent the absorbance values of the sample, negative control (PBS) and positive control (deionized water) groups, respectively.

### Cell viability assay

The hydrogel samples were pre-sterilized *via* ultraviolet irradiation for 24 hours, followed by incubation in DMEM to yield the hydrogel extract. HUVECs were plated in 96-well plates at a seeding density of 5000 cells per well. The cultured cells were treated with various extracts for 1, 2, and 3 days. After each treatment, the medium was replaced with complete medium containing Cell Counting Kit-8 (CCK-8) and incubated for 2 h. Absorbance was measured at 450 nm using a microplate reader (MULTISKAN GO). Cell viability was calculated as:5
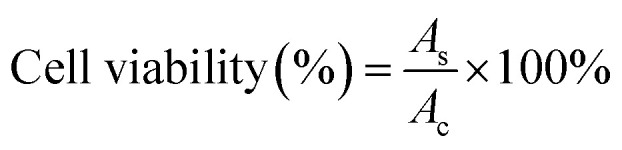
where *A*_s_ and *A*_c_ represent the absorbance values of the sample group and the control group, respectively.

Additionally, the samples following 1, 2, and 3 days of treatment were rinsed with PBS. Subsequently, a mixed staining solution consisting of calcein-AM, PI, and the corresponding buffer was uniformly dispensed onto the sample surfaces. The stained samples were then incubated in the dark at ambient temperature for 20–30 min, after which imaging was performed under a fluorescence microscope to capture fluorescent signals.

### ABTS radical scavenging assay

The antioxidant activity of the hydrogels was evaluated using the ABTS radical scavenging assay. Briefly, ABTS solution (8 mg mL^−1^, 25 mL) was mixed with potassium persulfate solution (1 mg mL^−1^, 25 mL) and incubated for 14 h to generate ABTS•^+^ radicals. Subsequently, hydrogel samples (200 mg) were mixed with 2 mL of the ABTS•^+^ solution and incubated in the dark at room temperature for 30 min. After incubation, the absorbance at 734 nm was recorded using a microplate reader. The ABTS radical scavenging rate was calculated according to the following equation:6
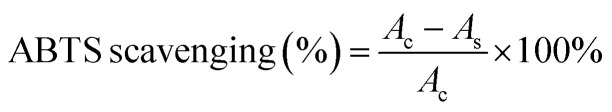
where *A*_c_ and *A*_s_ represent the absorbance values of the control and sample groups, respectively.

### Intracellular ROS scavenging and cell migration assays

Culture media supplemented with varying concentrations of H_2_O_2_ were added to 96-well plates pre-seeded with HUVECs. The H_2_O_2_ concentration corresponding to 50% cell viability (IC_50_) was determined *via* the CCK-8 assay (Fig. S4). For subsequent experiments, the culture medium in the 96-well plates was aspirated, after which 100 µL of medium containing 800 µM H_2_O_2_ was added, followed by incubation at 37 °C for 1 h to induce oxidative stress. Upon completion of H_2_O_2_ treatment, equal volumes of hydrogel extract were introduced into respective experimental groups, with continuous incubation for an additional 24 h. After the incubation period, the absorbance of each well at a wavelength of 450 nm was measured using a microplate reader for cell viability quantification.

Intracellular ROS levels were measured with a DCFH-DA probe. Cellular oxidative stress was induced *via* H_2_O_2_ treatment, and the stressed cells were co-incubated with hydrogel extract using the identical protocol described previously. The cells were then rinsed with PBS, after which a calibrated volume of DCFH-DA working solution was added. Following a 30 min incubation in a 37 °C incubator, the cells were washed again with PBS to remove excess staining reagent, and fluorescent microscopic imaging was performed to visualize intracellular ROS levels.

Under a laminar flow hood, three parallel reference lines were drawn on the bottom of each 6-well plate using a sterile marker pen. HUVECs were then seeded into the 6-well plates and cultured in complete medium for 24 h. A sterile 200 µL pipette tip was used to gently and uniformly scrape across the cell monolayer to create a consistent scratch. Following oxidative stress induction *via* H_2_O_2_-containing medium, the scratched cells were co-incubated with serum-free hydrogel extract for an additional 24 h. Microscopic images of the scratch regions were acquired at 0 h (immediately after scratch creation) and 24 h post-treatment, and cell migratory capacity was quantified using the following formula:7
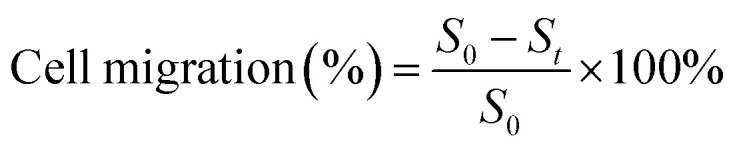
where *S*_0_ and *S*_*t*_ represent the scratch areas at 0 and 24 h, respectively.

### Evaluation of colorimetric pH response

SF/HA/Rhe hydrogels were exposed to 3 mL of PBS solutions with different pH values. After 2 h, color changes were observed and photographed. The RGB values of the images were analyzed using Image J software.

### Statistical analysis

All data are presented as the mean ± standard error (SE) from three independent experiments (*n* = 3). Statistical differences among groups were analyzed using one-way ANOVA, with significance levels set at *p < 0.05, **p < 0.01, and ***p < 0.001.

## Results and discussion

### Preparation and characterization of SF/HA/Rhe hydrogels

**Fig. 2:**
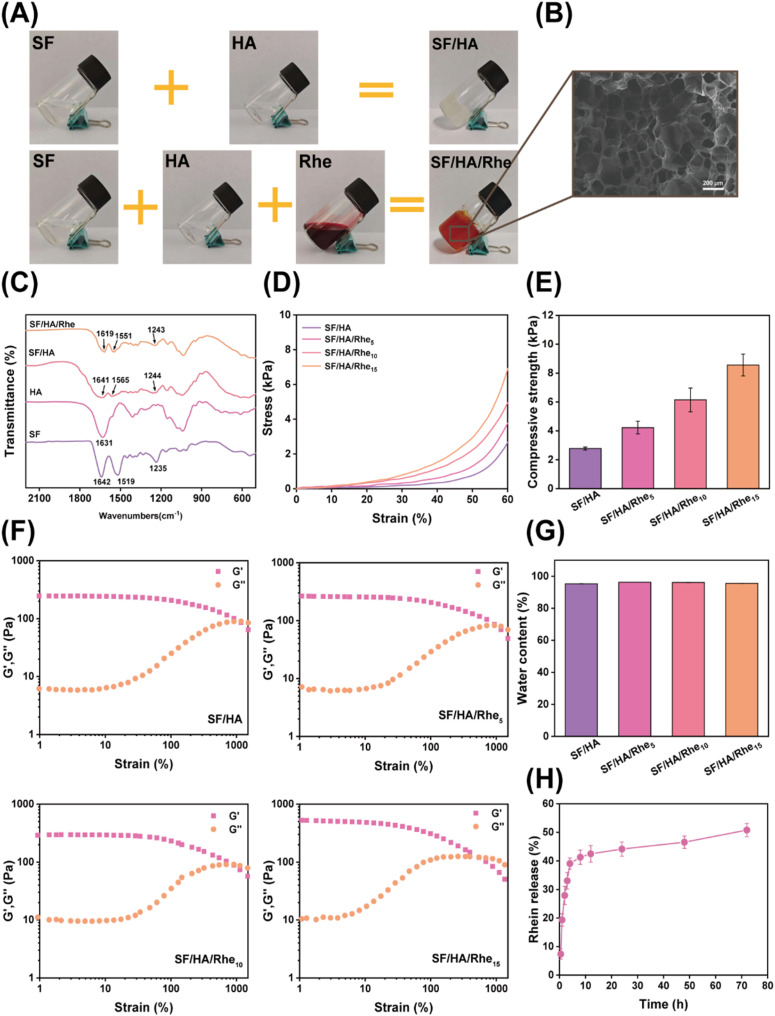
Preparation and characterization of SF/HA/Rhe hydrogels. (A) Gelation process of the SF/HA and SF/HA/Rhe hydrogels. (B) Microstructure of the SF/HA/Rhe hydrogel. (C) Fourier transform infrared spectra of SF, HA, SF/HA and SF/HA/Rhe. (D) Stress–strain curves of the hydrogels at 0–60% strain. (E) Compressive strength of the hydrogels. (F) Strain scan test of the hydrogels. (G) Water content of the hydrogels. (H) Rhe release from the hydrogel.

SF/HA and SF/HA/Rhe hydrogels were prepared through EDC/NHS-mediated crosslinking, in which SF and HA formed a covalently crosslinked network and Rhe was introduced as a functional component. The incorporation of Rhe imparted a distinct red coloration to the SF/HA/Rhe hydrogels (Fig. 2A). SEM observation showed that the SF/HA/Rhe hydrogel exhibited a porous three-dimensional microstructure (Fig. 2B). FTIR spectroscopy was used to analyze the characteristic functional groups and intermolecular interactions in SF, HA, SF/HA and SF/HA/Rhe (Fig. 2C). Pure SF exhibited characteristic absorption bands at 1642 cm^−1^ (amide I, random coil), 1519 cm^−1^ (amide II, β-sheet), and 1235 cm^−1^ (amide III), whereas HA displayed a characteristic band at 1631 cm^−1^, which was assigned to the asymmetric stretching vibration of carboxylate groups (COO^−^).^11^ After hydrogel formation, the amide II band shifted from 1519 cm^−1^ in pure SF to 1565 cm^−1^ in SF/HA and 1551 cm^−1^ in SF/HA/Rhe, indicating that the incorporation of HA and Rhe altered the local conformational environment of SF and the intermolecular interactions within the hydrogel network.^7,30^ In addition, the band in the amide I region shifted from 1641 cm^−1^ in SF/HA to 1619 cm^−1^ in SF/HA/Rhe, suggesting that Rhe incorporation altered the local molecular environment of the SF/HA network. These subtle spectral variations suggest intermolecular interactions among SF, HA and Rhe, including hydrogen bonding, which may help stabilize the hydrogel network.

Mechanical strength is important for the structural stability of hydrogel dressings. Therefore, the compressive behavior of the hydrogels was evaluated. The compressive strengths at 60% strain of SF/HA, SF/HA/Rhe_5_, SF/HA/Rhe_10_, and SF/HA/Rhe_15_ were 2.78, 4.23, 6.14 and 8.56 kPa, respectively (Fig. 2D and E). The compressive strength increased progressively with increasing Rhe content. Strain-sweep rheology further revealed the viscoelastic stability of the hydrogels. All Rhe-containing hydrogels exhibited higher storage moduli (*G*′) than the SF/HA, and SF/HA/Rhe_15_ showed the highest *G*′ among all groups (Fig. 2F). In contrast, the critical strain gradually decreased from SF/HA (1108%) to SF/HA/Rhe_5_ (1004%), SF/HA/Rhe_10_ (877%) and SF/HA/Rhe_15_ (536%). This trend suggests that increasing Rhe content enhanced the rigidity of the hydrogel network but reduced the critical strain. This behavior may be associated with additional noncovalent interactions introduced by Rhe, particularly hydrogen bonding between Rhe and the SF/HA matrix, which could strengthen intermolecular associations within the network.^31^

Swelling behavior and porosity are important for hydrogel dressings because they are closely related to fluid uptake and exudate management.^32^ As shown in Fig. S1(A), SF/HA exhibited a swelling ratio of 2128%, while the swelling ratios of SF/HA/Rhe_5_, SF/HA/Rhe_10_, and SF/HA/Rhe_15_ were 1987%, 1681% and 1448%, respectively. The corresponding porosity values were 73.8%, 67.8%, 65.5% and 60.6% for SF/HA, SF/HA/Rhe_5_, SF/HA/Rhe_10_ and SF/HA/Rhe_15_, respectively (Fig. S1B). The decrease in swelling ratio and porosity after Rhe incorporation was consistent with strengthened intermolecular interactions and a denser hydrogel network. Meanwhile, all hydrogels exhibited water contents above 95% (Fig. 2G), with values of 95.3% (SF/HA), 96.3% (SF/HA/Rhe_5_), 96.1% (SF/HA/Rhe_10_) and 95.6% (SF/HA/Rhe_15_). These results indicate the presence of a highly hydrated network, which is beneficial for maintaining a moist environment. In addition, the stability of the SF/HA/Rhe hydrogels in different media was evaluated by immersion in various solutions (Fig. S2). The hydrogels maintained their overall shape at all examined time points, indicating favorable structural stability. Meanwhile, the gradual coloration of the surrounding media during incubation visually suggested the progressive release of Rhe from the hydrogel, which was corroborated by the release profile shown in Fig. 2H. Rhe release was quantified in PBS at 37 °C using hydrogel samples of equal initial mass. As shown in Fig. 2H, the hydrogels exhibited an initial burst release of Rhe within the first 8 h, followed by a sustained release phase over 8–72 h. The initial burst release may result from the rapid diffusion of surface-associated or loosely retained Rhe, whereas the subsequent sustained release is likely related to the gradual diffusion of Rhe distributed within the hydrogel network and influenced by possible intermolecular interactions with the SF/HA matrix.^33,34^

### Antibacterial activity of SF/HA/Rhe hydrogels

**Fig. 3:**
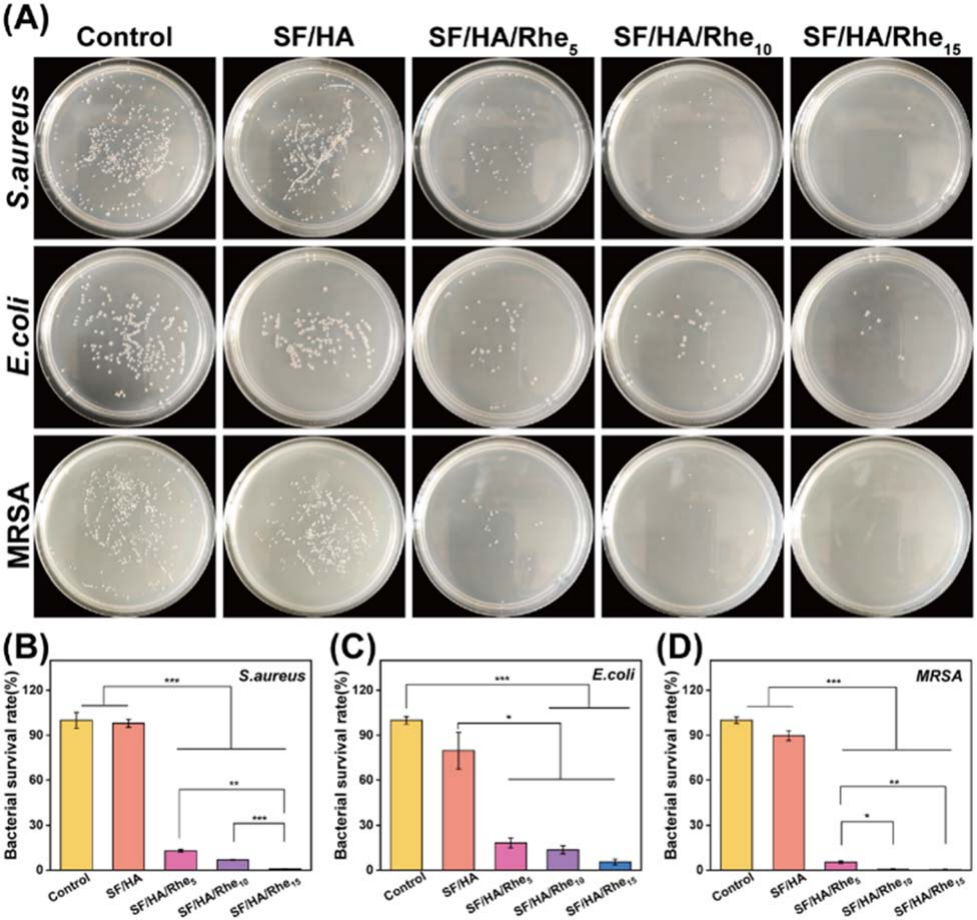
*In vitro* antibacterial properties of SF/HA/Rhe hydrogels. (A) Representative images of the antibacterial effects of SF/HA, SF/HA/Rhe hydrogels against *S. aureus*, *E. coli* and MRSA. Survival of *S. aureus* (B), *E. coli* (C) and MRSA (D) after hydrogel treatment. Quantitative data are presented as mean ± SE (*n* = 3). *p < 0.05, **p < 0.01, and ***p < 0.001.

Bacterial infection is a major factor that delays wound repair.^35,36^ The *in vitro* antibacterial activity of the SF/HA/Rhe hydrogels was evaluated by a colony-counting assay against representative wound-associated pathogens, including *S. aureus*, *E. coli* and MRSA. As shown in Fig. 3A, co-incubation with the SF/HA/Rhe hydrogels markedly reduced bacterial colony formation compared with the control group. Quantitative analysis of bacterial survival rates (Fig. 3B–D) showed that treatment with SF/HA/Rhe_5_ reduced the survival rates of *S. aureus*, *E. coli* and MRSA to 13.00%, 18.2%, and 5.4%, respectively. The antibacterial activity further increased with Rhe content, and SF/HA/Rhe_15_ exhibited the strongest effect, reducing the survival rates of *S. aureus*, *E. coli*, and MRSA to 0.82%, 5.47% and 0.27%, respectively. These results demonstrate that Rhe incorporation enhanced the antibacterial performance of the hydrogels in a concentration-dependent manner. This effect may be related to the intrinsic antibacterial activity of Rhe, which has been reported to disrupt bacterial membrane integrity and thereby reduce bacterial viability.^37^

### Biocompatibility of SF/HA/Rhe hydrogels

**Fig. 4:**
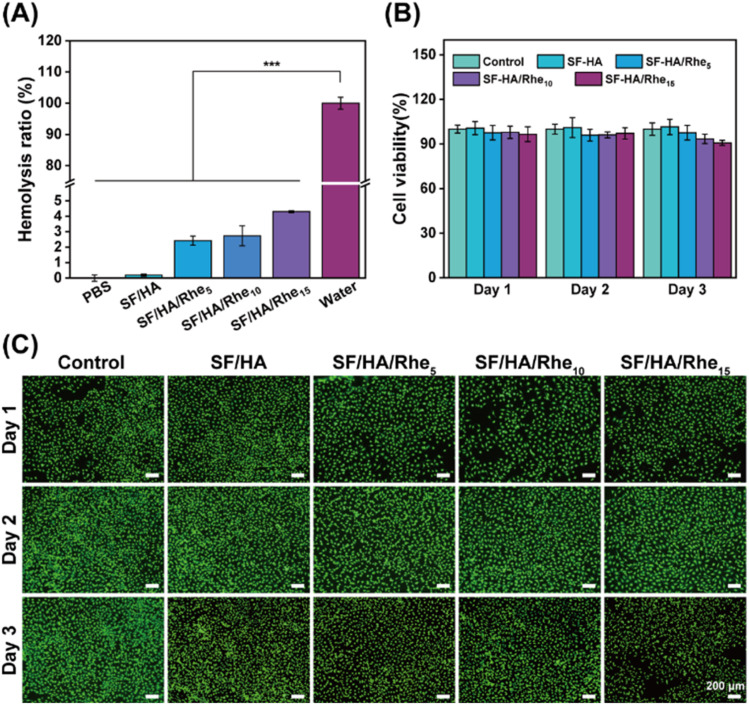
Biocompatibility of SF/HA/Rhe hydrogels. (A) Hemolysis rate measurement of SF/HA/Rhe hydrogels. (B) Cell viability tests of HUVECs treated by the hydrogel extracts. (C) Representative live/dead fluorescence images of hydrogel-treated HUVECs. Quantitative data are presented as mean ± SE (*n* = 3). ***p < 0.001.

The biocompatibility of the hydrogels was first assessed by a hemolysis assay.^38,39^ SF/HA and SF/HA/Rhe hydrogels were incubated with red blood cell (RBC) suspension, and no evident hemolysis was observed in either group, with a response comparable to that of the PBS control (Fig. S3). Quantitative analysis showed that the hemolysis rates of all hydrogel groups were below 5% (Fig. 4A), indicating good hemocompatibility. The cytocompatibility of the SF/HA/Rhe hydrogels was further evaluated using HUVECs exposed to hydrogel extracts. Cell viability was determined by CCK-8 assay after 1, 2 and 3 days of incubation. As shown in Fig. 4B, cell viability remained above 90% in all groups treated with hydrogel extracts at each time point, indicating negligible cytotoxicity. Consistently, live/dead staining revealed predominantly viable cells in all SF/HA/Rhe hydrogel groups, with no apparent difference from the control group (Fig. 4C). These findings demonstrate the favorable hemocompatibility and cytocompatibility of the SF/HA/Rhe hydrogels.

### Antioxidant capacity of SF/HA/Rhe hydrogels

**Fig. 5:**
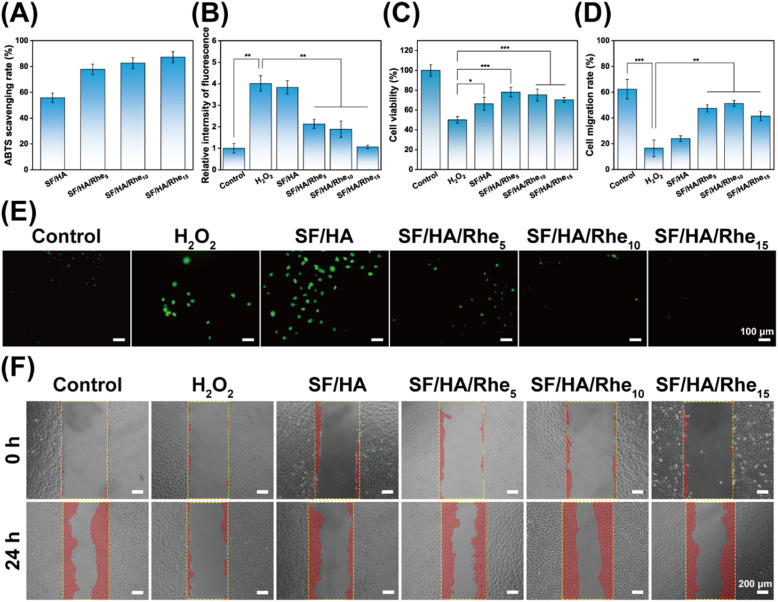
Antioxidant properties of SF/HA/Rhe hydrogels. (A) Scavenging rate of ABTS by hydrogels. (B) Relative fluorescence intensity of HUVECs incubated with SF/HA/Rhe hydrogels for 24 h after H_2_O_2_ treatment. (C) Cell viability of HUVECs incubated with SF/HA/Rhe hydrogels for 24 h after H_2_O_2_ treatment. (D) Migration of HUVECs under different treatments. (E) Images of HUVECs under different treatments detected by DCFH-DA probe. (F) Microscopic images of migration of HUVECs under different treatments at 0 h and 24 h. Quantitative data are presented as mean ± SE (*n* = 3). *p < 0.05, **p < 0.01, and ***p < 0.001.

Excessive ROS accumulation at infected wound sites can aggravate oxidative stress and impair wound healing.^40^ Therefore, effective ROS scavenging capacity is an important functional requirement for hydrogel dressings. The antioxidant activity of the hydrogels was evaluated using an ABTS radical scavenging assay. Fig. 5A shows that Rhe incorporation significantly enhanced the ABTS radical scavenging activity of the hydrogels, and the scavenging capacity further increased progressively with Rhe content. Among all groups, SF/HA/Rhe_15_ exhibited the strongest activity, with a scavenging rate of 87.22%. This antioxidant effect is likely associated with the chemical structure of Rhe. As a hydroxyanthraquinone, Rhe may scavenge radicals through hydrogen or electron donation from its phenolic hydroxyl groups, while its conjugated anthraquinone scaffold may help stabilize the resulting radical species.^41,42^

The intracellular ROS-scavenging capability of the hydrogels was further evaluated in HUVECs under H_2_O_2_-induced oxidative stress. Representative fluorescence images and corresponding quantitative analysis (Fig. 5B and E) showed that H_2_O_2_-treated cells exhibited markedly stronger green fluorescence than the negative control, indicating substantial intracellular ROS accumulation. In contrast, treatment with SF/HA/Rhe hydrogel extracts reduced the fluorescence intensity, and this reduction became more pronounced with increasing Rhe content. In addition, cell viability under oxidative stress was evaluated after exposure to hydrogel extracts (Fig. 5C). Compared with the H_2_O_2_ group (50.08%), all SF/HA/Rhe extract-treated groups exhibited improved cell viability, with values exceeding 70%. These results indicate that SF/HA/Rhe hydrogels can alleviate intracellular ROS accumulation and protect HUVECs against H_2_O_2_-induced oxidative injury.

To further evaluate whether the hydrogels could restore cell migration impaired by oxidative stress, a scratch assay was performed. Representative images and quantitative analysis (Fig. 5D and F) showed that cells treated with H_2_O_2_ alone exhibited minimal scratch closure, with a migration rate of only 16.50% after 24 h. In contrast, treatment with SF/HA/Rhe hydrogel extracts significantly improved the migratory capacity of H_2_O_2_-treated cells, and the SF/HA/Rhe_10_ group reached a migration rate of 51.26%. This improvement in migration was consistent with the antioxidant and cytoprotective effects of the hydrogels under oxidative stress. Since excessive intracellular ROS may impair cellular function, the reduced ROS accumulation observed after treatment with SF/HA/Rhe hydrogel extracts is likely one of the factors contributing to the restoration of HUVEC migratory behavior.^43^ Together with the improved cell viability, these results suggest that the ROS-scavenging effect of the hydrogels may help preserve cellular function and support migration recovery.

### Colorimetric pH response of SF/HA/Rhe hydrogels

**Fig. 6:**
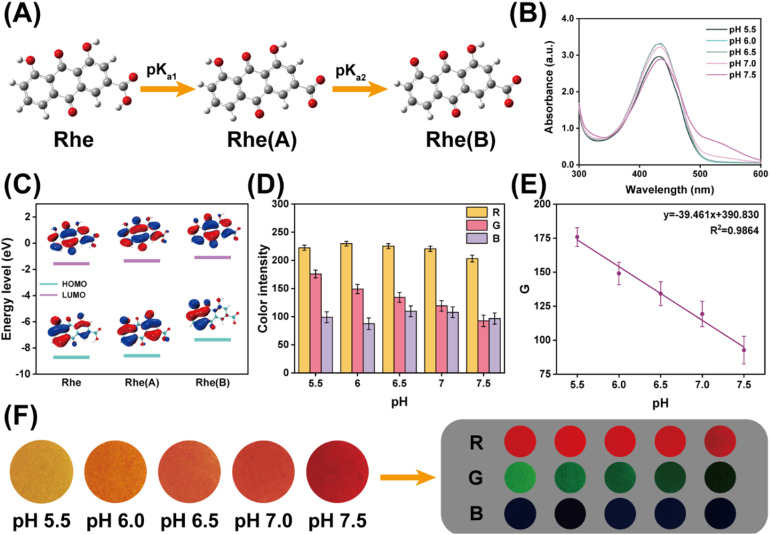
Colorimetric pH response of SF/HA/Rhe hydrogel. (A) Three protonated states of Rhe. (B) UV-visible spectra of Rhe solution under different pH conditions. (C) HOMO–LUMO energy level diagram of Rhe with different protonation states. (D) Average *R*, *G* and *B* values of SF/HA/Rhe hydrogel images under different pH conditions. (E) Linear correlation plot of the *G*-channel intensity of SF/HA/Rhe hydrogel images *versus* pH value. (F) Diagrams of SF/HA/Rhe hydrogels under different pH conditions.

Since changes in wound-site pH can provide important information regarding bacterial infection and healing progression, the pH-responsive colorimetric behavior of Rhe was first investigated.^23^ Rhe was first dissolved in deionized water under mildly alkaline conditions to improve its solubility, and the resulting alkalized solution was then added dropwise into buffer solutions of different pH values. As shown in Fig. S5, the color of the resulting solutions gradually changed from yellow to deep red with increasing pH, indicating a clear pH-dependent optical response. This color evolution is consistent with the pH-dependent acid–base equilibrium of Rhe, in which stepwise deprotonation occurs first at the carboxyl group and subsequently, at higher pH, at the phenolic hydroxyl group (Fig. 6A).^44^ In agreement with this interpretation, the UV-vis spectra showed a gradual decrease in the absorption band at 435 nm together with a progressive increase in the band at 505 nm as pH increased (Fig. 6B), indicating a pH-dependent shift in the relative distribution of Rhe species. Furthermore, HOMO–LUMO calculations showed that the deprotonated Rhe(B) form possessed a smaller energy gap than Rhe(A) (Fig. 6C).^45^ This result supports the view that deprotonation enhances electron delocalization within the conjugated structure of Rhe, thereby lowering the transition energy and promoting absorption at longer visible wavelengths.

The pH-responsive colorimetric behavior of the SF/HA/Rhe hydrogels was then evaluated. As shown in Fig. 6F, exposure of the hydrogel surface to buffer solutions of different pH values induced a gradual color transition from yellow to deep red, indicating that the pH-responsive optical behavior of Rhe was retained after incorporation into the hydrogel matrix. To quantify this visual response, RGB analysis of the hydrogel images was performed using ImageJ by separating the red, green, and blue channels and calculating the mean intensity of each channel (Fig. 6D). Among the three channels, the *G*-channel exhibited the most pronounced and monotonic variation with pH, whereas the *R*-channel remained relatively stable and the *B*-channel showed no distinctive trend. In particular, the *G*-channel intensity gradually decreased with increasing pH and displayed a strong linear correlation over the pH range of 5.5–7.5, following the equation *G* = −39.461 pH + 390.830 (Fig. 6E). This relationship suggests the feasibility of semi-quantitative pH estimation within the tested interval. Collectively, these results demonstrate that the SF/HA/Rhe hydrogels possess distinct pH-responsive colorimetric behavior and hold promise for visual indication of wound microenvironmental pH changes.

## Conclusions

In this study, a multifunctional SF/HA/Rhe hydrogel was successfully developed by incorporating Rhe into an SF/HA network through a facile fabrication strategy. The resulting hydrogels exhibited improved mechanical performance, favorable biocompatibility and enhanced antibacterial and antioxidant activities. Notably, the SF/HA/Rhe hydrogels showed effective ABTS radical scavenging activity, alleviated intracellular ROS accumulation and improved cell migration under oxidative stress. In addition, the hydrogels displayed distinct pH-responsive colorimetric behavior, highlighting the feasibility of using Rhe as a functional component for visual pH indication. Overall, the SF/HA/Rhe hydrogel represents a promising smart wound dressing candidate that integrates antibacterial, antioxidant, and pH-responsive colorimetric functions, with potential for antibacterial intervention and visual monitoring of pH changes in the wound microenvironment.

## Author contributions

Miaoshen Kou: conceptualization, methodology, data curation, visualization, writing—original draft preparation. Deng Wang: investigation, data curation, visualization, writing—original draft preparation. Jiawei Zhang: data curation. Fangzheng Yu: validation. Yang Yuan: investigation. Chen Wang: visualization. Jiale He: writing—review and editing. Zheng Zhao: formal analysis, writing—review and editing, supervision, project administration. Yichao Liu: writing-review and editing, supervision, project administration.

## Conflicts of interest

All authors declare that they have no conflicts of interest.

## Supplementary Material

RA-016-D6RA03047E-s001

## Data Availability

The data that support the findings of this study are available from the corresponding author upon reasonable request. The data supporting the findings of this study are available within the article and its supplementary information (SI). Supplementary information: additional experimental results and supporting figures. See DOI: https://doi.org/10.1039/d6ra03047e.
