# Supplementary material for: A rhein-integrated hydrogel dressing with antibacterial, antioxidant and pH-responsive colorimetric properties
Source: RSC Adv. 2026 May 22;16(31):28092–102. doi: 10.1039/d6ra03047e (PMC13202421; doi:10.1039/d6ra03047e)
Supplement: RA-016-D6RA03047E-s001 [file RA-016-D6RA03047E-s001.pdf]

Supplementary Information (SI) for RSC Advances.
This journal is © The Royal Society of Chemistry 2026

Supplementary Information

A Rhein-Integrated Hydrogel Dressing with Antibacterial, Antioxidant and pH-Responsive Colorimetric Properties

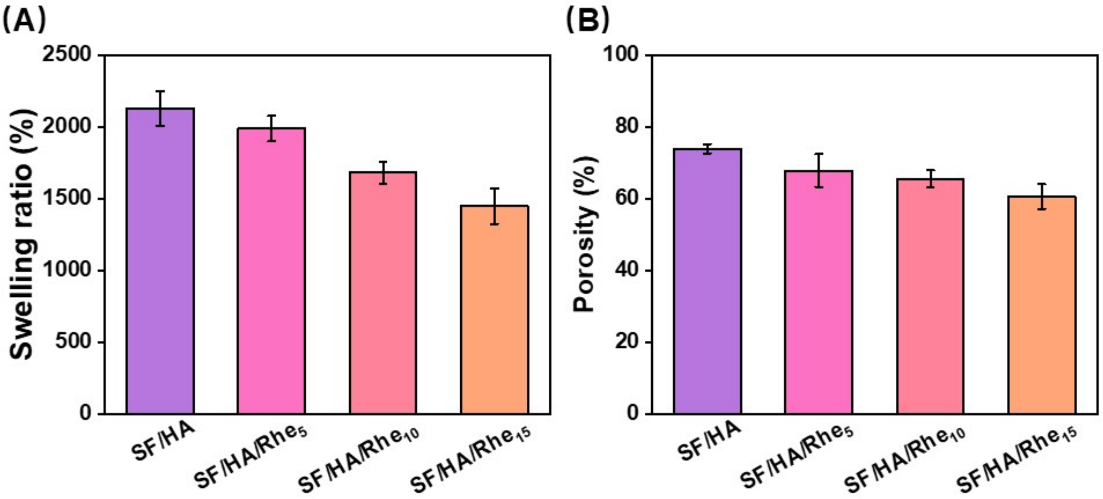

Figure S1. Swelling properties of SF-HA/Rhe hydrogels.

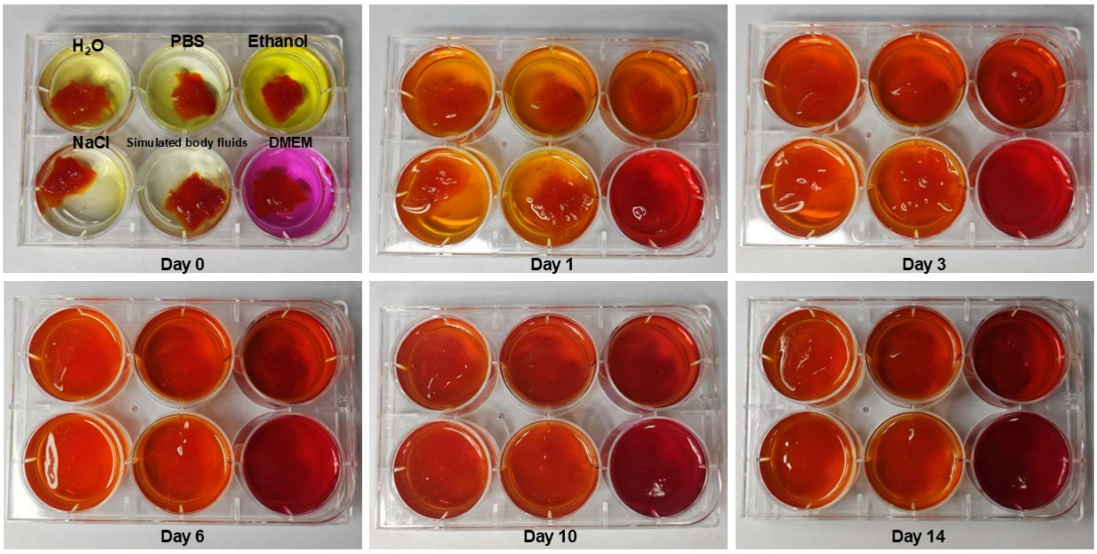

Figure S2. Corresponding images of SF-HA/Rhe hydrogels immersed in different solutions on days 0, 1, 3, 6, 10, and 14.

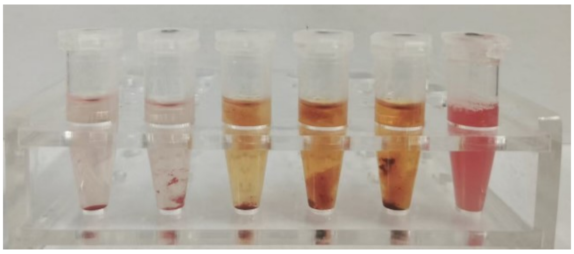

Figure S3. Hemolysis photographs of the hydrogels (from left to right: PBS group, SF-HA group, SF-HA/Rhe_5, SF-HA/Rhe_{10} and SF-HA/Rhe_{15} hydrogel groups and water group, respectively).

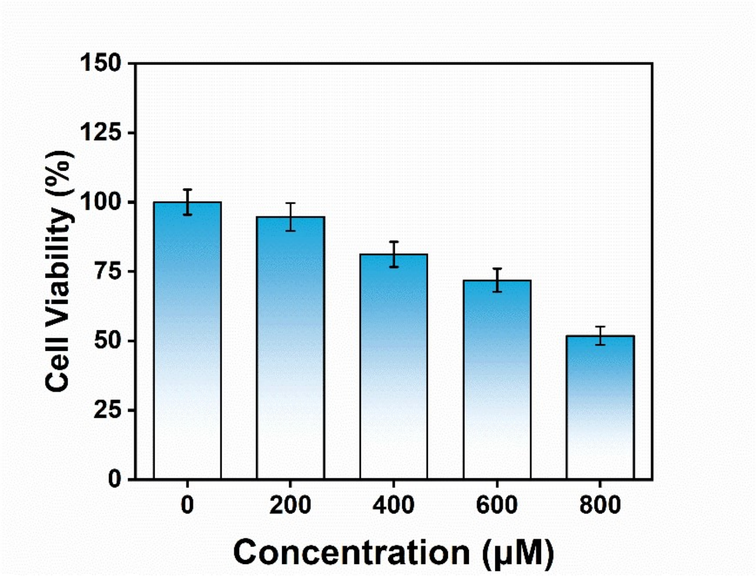

Figure S4. Cell viability of HUVECs treated with different concentrations of H_2O_2 for 1 hour.

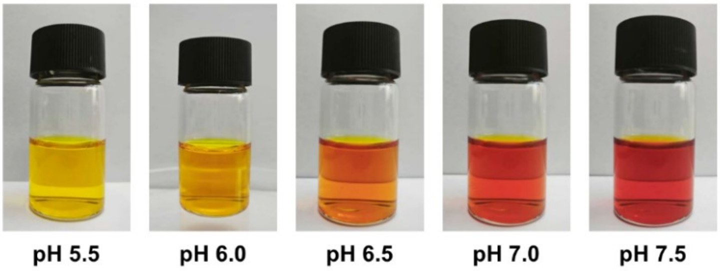

Figure S5. Diagrams of Rhe solutions under different pH conditions.
